# Resilience and problematic smartphone use: a moderated mediation model

**DOI:** 10.1186/s12888-023-04541-1

**Published:** 2023-01-13

**Authors:** Zejun Hao, Liangyi Jin, Jinzi Huang, Hafiza Rabia Akram, Qian Cui

**Affiliations:** 1 Institute of Foreign Languages, China Medical University, No.77 Puhe Road, Shenyang North New Area, Shenyang, Liaoning Province 110122 People’s Republic of China; 2 Shenyang Women’s and Children’s Hospital, No.87 Danan Street, Shenhe District, Shenyang, Liaoning Province 110011 People’s Republic of China; 3 Liaoning National Normal College, No. 45, Chongshan East Road, Huanggu District, Shenyang, Liaoning Province 110032 People’s Republic of China; 4 China Medical University, No.77 Puhe Road, Shenyang North New Area, Shenyang, Liaoning Province 110122 People’s Republic of China; 5 Department of Rehabilitation, Shengjing Hospital of China Medical University, No. 16 Puhe Road, Shenyang North New Area, Shenyang, Liaoning Province 110134 People’s Republic of China

**Keywords:** Resilience, Perceived social support, The sense of school belonging, Habitual smartphone use, Problematic smartphone use

## Abstract

**Background:**

Problematic smartphone use (PSU) is adversely correlated with resilience. To completely comprehend the mechanism underlying this relationship, however, more investigation is required.

**Methods:**

For this cross-sectional study, the method of stratified random cluster sampling was applied. 834 Chinese undergraduate students (aged 17 to 24) were recruited, and self-reported questionnaires were administered to measure their levels of resilience, perceived social support, the sense of school belonging, and habitual and problematic smartphone use.

**Results:**

The findings showed that resilience both directly and indirectly predicted PSU through perceived social support and the sense of school belonging. Additionally, there were significant moderating effects of habitual smartphone use between resilience and perceived social support, the sense of school belonging, and PSU.

**Conclusions:**

Our research identified the negative influence of resilience on PSU, and specifically, highlighted the mediation effects of perceived social support and the sense of school belonging. Of significance, we also found the moderation effect of habitual smartphone use in the development of PSU.

## Background

With the development of communication technology, using a smartphone has become a necessary component of a person’s everyday life. By June 2022, there have been 104.7 million smartphone internet users in China [1]. Along with its advantages of instant communication and productivity enhancement, excessive smartphone use has been reported to induce the physical impairments and mental health issues of the users. Of note, this scenario is even more obvious among the youth population who are subject to higher occurrence of smartphone overuse [2, 3]. Therefore, there has been a request to investigate the issue of smartphone overuse and discover the mechanism that leads to it. The current study focused on the association between resilience and problematic smartphone use (PSU). Additionally, we looked at the mediation effects of perceived social support and the sense of school belonging and the moderation effect of habitual smartphone use in the progression of PSU.

Problematic smartphone use (PSU) represents the uncontrollable use of smartphone which causes the users to lose some of their functionalities [4, 5]. PSU relates to symptoms similar to those of behavioral addictions such as shoulder discomfort [6], insomnia [7], and hand dysfunction [8]. Those who engage in PSU are more likely to endure loneliness [9], interpersonal issues [10], and distress [11]. In addition, students in college who use their smartphones problematically often perform worse academically [12]. The I-PACE theory [13, 14] has proposed the process underpinning the development of problematic smartphone and a significant pathway is that individuals choose to overuse their smartphones to get rid of the unwanted feelings [15]. Furthermore, greater levels of alexithymia [16], and lower self-control [17] and self-esteem [18] also contribute to PSU.

Resilience refers to the capability to endure hardships in life, to bounce back from trauma and catastrophe, and to adapt successfully [19–21]. Resilience is linked with an array of positive psychological traits including optimism, a high threshold for negative emotions, and self-reflection [22, 23]. Resilience is also effective for attenuating the anxiety and despair symptoms during the pandemic [24, 25]. Furthermore, resilience improves people’s ability to manage their urges and lowers their risk of engaging in harmful behaviors. Therefore, resilient persons are observed with fewer drug usage [26], gambling addiction [27], and other addictive behaviors [28]. Based on this, we made the assumption that people with greater resilience should be better able to withstand the urge to use their smartphone excessively.

The Broaden-and-Build theory [29] contends that resilience encourages a person to accumulate more beneficial psychological resources. Therefore, those who are more resilient are also seen to have higher levels of perceived social support and the sense of school belonging [30, 31]. Perceived social support refers to the feeling of being supported by families and friends and it assists a person to improve their psychological wellness [32]. A person who has social support is less likely to be negatively impacted by negative emotions and instead maintains mental clarity to attend to the pressing demands [33, 34]. Resilient individuals excel in maintaining positive bonds with their family and friends [19]. As a result, these individuals could readily rely on their local surroundings for help. Additionally, the adequate social support enables one to gain strength through trying times [30] and become less likely dependent on the excessive smartphone use for extra resources. This quality might help prevent PSU and other addictive behaviors to some extent [35, 36].

The sense of school belonging describes students’ feelings of acceptance, respect, and support in the educational environment [37]. A strong sense of school identity makes people feel more supported, less lonely, and increases their belief in life [38]. As a result, they might retain psychological equilibrium and be less prone to experience despair and anxiety [39]. Resilience helps a person to be adapted to an environment and thus promotes an individual to achieve the sense to belong [40]. In schools, resilient students are more motivated to participate in the school activities and develop healthy interpersonal relationship with teachers and classmates [31]. Further, a strong sense of school identity lessens a student’s need to rely on their smartphone to establish positive relationships with other students, which lowers their risk of developing PSU [41]. Together, we proposed the hypothesis that perceived social support and the sense of school belonging should act as mediators between resilience and PSU.

Habitual behaviors are repeated actions that are prompted by situational circumstances and occur without self-instruction or deliberate purpose [42, 43]. According to this definition, habitual smartphone use describes the automatic activation of a smartphone by internal or external stimuli [43, 44]. The COVID-19 pandemic has intensified the smartphone use [45] in that a person has to rely on the virtual communication available through smartphone use to stay current with the state of affairs and get resources from the outside. With the continuous pandemic, the prompted automatic smartphone use progressively develops into a habit to receive support and satisfy the need to belong. This is particularly true of the resilient persons who are always prepared to seek resources from the immediate surroundings by using handy equipment. Therefore, we hypothesized that habitual smartphone use should moderate between resilience and PSU, perceived social support, and the sense of school belonging. With the more habitual smartphone use, each separate impact should become stronger.

**Fig. 1:**
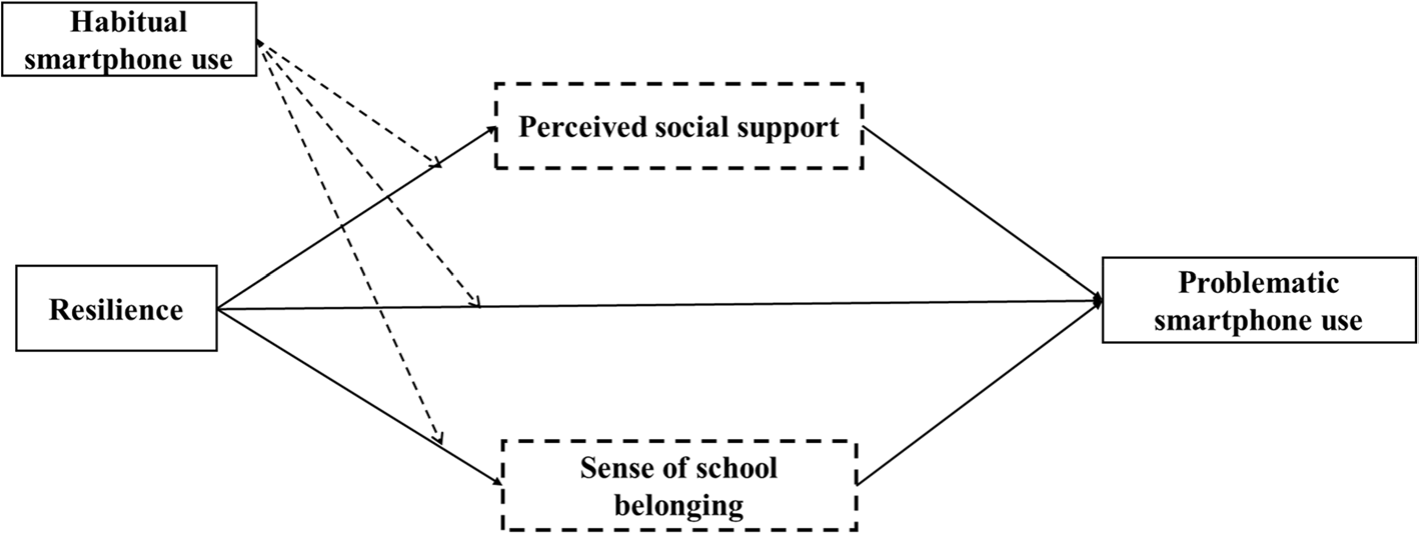
The hypothesized model. The dotted boxes contained the investigated mediators and the dotted lines indicated the investigated moderation effects

According to the Broaden-and-Build theory [29], a resilient people would seek resources to deal with the ongoing difficulties. In this regard, such students would very likely look to their immediate surroundings such as the school and community for support by using their smartphones. Given the aforementioned, we hypothesized: 1) resilience should negatively predict PSU; 2) this relationship should be mediated by perceived social support and the sense of school belonging; 3) habitual smartphone use should moderate between resilience and PSU, the sense of school belonging, and perceived social support. The proposed model is shown in Fig. 1.

## Methods

### Participants and procedure

We selected the participants by the method of stratified random cluster sampling from two universities in Liaoning Province, located in the Northeast China. In May 2020, students were undergoing home quarantine and continuing their studies through virtual classes. Therefore, an online survey was carried out. Digital surveys were created and stored in a website (https://www.wjx.cn/), an online platform widely used for collecting data in China. The respondents were sent with the link at the end of their online courses and completed the questions. The participants in this study majored in a range of subjects, including linguistics, electrical engineering, and art education, among others. The inclusion criteria were Chinese undergraduate students who were fully capable of reading and comprehending the survey questions as well as being able to finish it on their own. The sample size was determined by referring to the principle of a subject-to-item ratio of at least 5:1 [46] and a minimum of 200 observations [47] . Prior research that tested the moderated mediation employed these criteria [48, 49]. The current final sample size was adequate to satisfy the requirements. A total of 871 Chinese undergraduates joined in the survey, and 834 provided reliable answers (resulting the response rate 95.75%). The participants’ average age was 20.09 ± 1.17 years (ranged between17 to 24). Male students (*n* = 188, 22.5%) participated in the survey at a lower rate than female students (*n* = 646, 77.5%). The assessment was approved by the Ethics Committee at Liaoning National Normal College. Before taking the survey, the participants were asked to read the instructions to gain a full understanding of the current study. Only after providing their informed consents, could the students start taking the survey. All the collected data were protected and could be accessed by the authors only. In conducting the study, the Declaration of Helsinki’s principles were followed.

### Measures

#### Smartphone addiction scale-short version (SAS-SV)

To determine the extent of PSU, the Chinese version of SAS-SV [50] was used. This 10-item scale is based on a 6-point Likert system with the options “1 = strongly disagree” and “6 = strongly agree.” Examples of items are “Having my smartphone on my mind even when I’m not using it” and “Feeling pain in the wrists or the back of the neck while using a smartphone.” The Chinese version of SAS-SV has been widely used in Chinese participants with a high level of reliability [51, 52]. In the current sample, the Cronbach’s alpha was 0.844.

#### The Connor-Davidson resilience scale (CD-RISC)

The degree of resilience was assessed using the Chinese version of CD-RISC [53] . The 25 items scale runs on the 5-point Likert system in which answers range from “0 = not true at all” to “4 = true nearly all the time.” Examples of items are “Able to adapt to change” and “In charge of your life,” among others. Many Chinese participants have been tested using the Chinese version of CD-RISC, and it has good validity [20, 54]. In the current sample, the Cronbach’s alpha was 0.932.

#### Perceived social support scale (PSSS)

The Chinese version of PSSS [55], which has 12 items, was used to gauge the participants’ perceptions of social support. Scores vary from 1 to 7, with 1 denoting a very strong disagreement and 7 denoting a very strong agreement. Items like “My family really strives to help me” and “I can count on my pals when things go wrong” are examples of such statements. The questionnaire has been used frequently by Chinese participants and its validity has been shown [20, 30]. In the current sample, the Cronbach’s alpha was 0.942.

#### Psychological sense of school membership scale (PSSM)

Using the Chinese version of PSSM, the feeling of belonging at school was evaluated [37]. 18 items make up the 6-point Likert scale, with 1 denoting “very strongly disagree” and 6 denoting “very strongly agree”. Items like “I felt like a real member of the school” and “People notice when I am good at something” are examples of such statements. The validity of the Chinese version of PSSM has been demonstrated to be high [56]. In the current sample, the Cronbach’s alpha was 0.901.

#### Habitual smartphone use scale (HSUS)

Habitual smartphone use was tested with a questionnaire developed in a previous study [44]. HSUS includes 6 items and runs on a 5 point-Likert system ranging from “1 = strongly disagree” to “5 = strongly agree”. “Smartphone is my everyday routine” and “It is a habit to utilize my smartphone” are examples of sample items. The Cronbach’s alpha in the current sample was 0.818.

#### Data analyses

The current study’s statistical analyses were performed using SPSS 22. The threshold for significance was fixed at 0.05. We first tested the correlations between the variables using the Pearson analysis. Second, by creating bias-corrected bootstrap confidence intervals, model 4 of SPSS macro PROCESS [57] was used to test the mediators of perceived social support and the sense of school belonging between resilience and PSU (using 5000 bootstrapping samples). Third, the moderating effects of habitual smartphone use between resilience and PSU, perceived social support, and the sense of school belonging were examined using model 8 of SPSS macro PROCESS [57]. Age and gender were treated as covariates in the analyses since they were substantially connected with PSU in previous studies [44, 58, 59].

## Results

### Correlations of the investigated variables

Resilience is positively correlated with perceived social support, a sense of school belonging, and habitual smartphone use, but negatively correlated with PSU (see Table 1).

**Table 1:** Correlations among the investigated variables

	M	SD	1	2	3	4	5
1. Resilience	88.56	14.019					
2. Social support	62.41	12.437	0.507***				
3. School belonging	77.56	12.321	0.45***	0.554***			
4. Habitual use	12.26	4.193	0.79*	−0.038	0.88*		
5. PSU	30.41	8.078	−0.291***	− 0.28***	− 0.297***	−0.381***	

*N* = 834. *PSU* Problematic smartphone use, *M* Mean, *SD* Standard deviations. *p<0.05, ***P < 0.001

### The mediators of perceived social support and the sense of school belonging

Table 2 shows that resilience was positively associated with perceived social support (β = 0.457, *p* < 0.001) and the sense of school belonging (β = 0.405, *p* < 0.001), and negatively associated with PSU (β = − 0.099, *p* < 0.001); in turn, perceived social support (β = − 0.066, *p* < 0.05) and the sense of school belonging (β = − 0.106, *p* < 0.001) were negatively related to PSU. The results of the mediation analyses revealed that resilience significantly and positively predicted PSU (β = − 0.172, *p* < 0.001) and indirectly through the sense of school belonging (= − 0.043, 95% confidence interval − 0.067 to − 0.019) and perceived social support (= − 0.03, 95% confidence interval − 0.055 to − 0.006) (see Table 2 &Table 3). Namely, the influence of resilience on PSU was partially mediated by perceived social support and the sense of school belonging.

**Table 2:** Mediation analysis by Process model 4

Outcome variable	Independent variables	β	SE	t	P
PSU	Constant	49.221***	4.958	9.928	0.000
	Age	−0.56	0.229	−0.245	0.807
	Gender^a^	−1.374*	0.645	−2.132	0.033
	Resilience	−0.172***	0.019	− 8.948	0.000
Social support	Constant	11.714	6.86	1.708	0.088
	Age	0.289	0.317	0.911	0.362
	Gender^a^	2.492**	0.892	2.974	0.005
	Resilience	0.457***	0.027	17.172	0.000
School belonging	Constant	37.787***	7.044	5.364	0.000
	Age	−0.03	0.326	−0.093	0.926
	Gender^a^	2.566**	0.916	2.802	0.005
	Resilience	0.405***	0.027	14.804	0.000
PSU	Constant	54.005***	4.941	10.929	0.000
	Age	−0.04	0.225	−0.178	0.858
	Gender^a^	−0.937	0.636	−1.474	0.141
	Social support	−0.066*	0.027	−2.456	0.014
	School belonging	−0.106***	0.026	−4.025	0.000
	Resilience	−0.099***	0.023	−4.372	0.000

*N* = 834. *PSU* Problematic smartphone use. *p<0.05, **p<0.01, ***p<0.001

^a^ Male = 1, female = 2

**Table 3:** Bootstrapping indirect effect and 95% confidence interval (CI) for the mediation model by Process model 4

Indirect path	Estimated effect	95% CI	
		Lower	Upper
Resilience → perceived social support → PSU	^a^-0.03	−0.055	−0.006
Resilience → school belonging → PSU	^a^-0.043	−0.067	−0.019

*N* = 834. *PSU* Problematic smartphone use. Bootstrap sample size = 5000. *CI* Confidence interval

^a^Empirical 95% confidence interval does not overlap with zero

### The moderator of habitual smartphone use

**Fig. 2:**
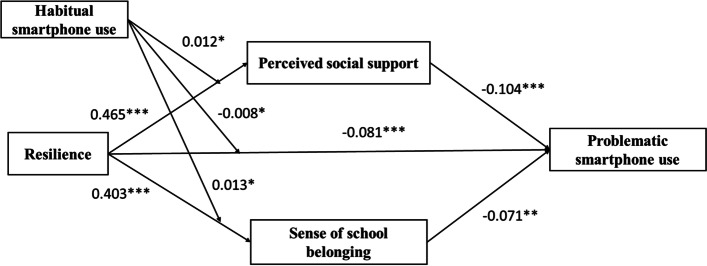
The verified model (*N* = 834). Habitual smartphone use significantly moderated the association between resilience and problematic smartphone use, perceived social support, and the sense of school belonging

Table 4 displays the outcomes of model 8 from SPSS macro PROCESS 3.1 [57]. Habitual smartphone use significantly moderated between resilience and PSU: The negative correlation between resilience and PSU got worse as habitual smartphone use increased (resilience × habitual smartphone use, β = − 0.008, *p* = 0.001, 95% confidence interval − 0.015 to − 0.001); habitual smartphone use significantly moderated between resilience and perceived social support: The positive relationship between resilience and perceived social support improved with increased resilience (resilience × habitual smartphone use, β = 0.012, *p* < 0.05, 95% confidence interval 0.001 to 0.022); habitual smartphone use moderated between resilience and the sense of school belonging: The correlation between resilience and a sense of belonging at school became stronger with higher level of habitual smartphone use (resilience × habitual smartphone use, β = 0.013, *p* < 0.01, 95% confidence interval 0.002 to 0.024) (see Fig. 2);

**Table 4:** The moderation analysis by Process model 8

Outcome variable	Independent variable	β	SE	t	P	BootLLCI	BootULCI
Perceived social support	Constant	50.366***	6.486	7.765	0.000	37.635	63.097
	Age	0.365	0.316	1.157	0.248	−0.255	0.985
	Gender^a^	2.619**	0.887	2.952	0.003	0.877	4.36
	Habitual smartphone use	−0.289**	0.091	−3.193	0.001	−0.467	−0.112
	Resilience	0.465***	0.027	17.511	0.000	0.413	0.517
	Resilience x habitual smartphone use	0.012*	0.005	2.161	0.031	0.001	0.022
Sense of school belonging	Constant	73.687***	6.677	11.036	0.000	60.581	86.792
	Age	−0.043	0.325	−0.131	0.896	−0.681	0.596
	Gender^a^	2.63**	0.913	2.88	0.004	0.837	4.423
	Habitual smartphone use	0.1	0.093	1.075	0.283	−0.083	0.283
	Resilience	0.403***	0.027	14.765	0.000	0.35	0.457
	Resilience x habitual smartphone use	0.013*	0.006	2.339	0.02	0.002	0.024
PSU	Constant	41.546***	4.582	9.068	0.000	32.533	50.539
	Age	0.122	0.207	0.588	0.557	−0.285	0.529
	Gender^a^	−0.859	0.586	−1.466	0.143	−2.008	0.291
	Habitual smartphones use	−0.678	0.06	−11.285	0.000	−0.796	−0.56
	Social support	−0.104***	0.025	−4.139	0.000	−0.153	−0.055
	Sense of school belonging	−0.071**	0.024	−2.919	0.004	−0.119	−0.023
	Resilience	−0.081***	0.021	−3.852	0.000	−0.122	−0.04
	Resilience x habitual smartphone use	−0.008*	0.004	−2.215	0.027	−0.015	−0.001

*N* = 834. *PSU* Problematic smartphone use, *p<0.05, **p<0.01, ***p<0.001

^a^Male = 1, female =2. Bootstrap sample size = 5000. *LL* Low limit, *CI* Confidence interval, *UL* Upper limit

## Discussion

The current study integrated resilience, perceived social support, sense of school belonging, habitual smartphone use, and PSU into a model. We investigated the mediation effects of perceived social support and the sense of school belonging and the moderation effects of habitual smartphone use in the association between resilience and PSU.

### The direct impact on PSU

Supporting hypothesis 1, resilience negatively predicted PSU and this concurs with the previous findings [60, 61]. A person who is resilient avoids the distractions of unfavorable life situations, which helps them reach a higher mental health state [52] [53].. The pandemic has increased people’s involvement with smartphone use, but a realistic viewpoint and a strong sense of self-control equip users with the right mindset for current difficulties and assist students in using smartphones in a healthy way [60]. Therefore, resilient people have fewer chances of developing PSU.

### The mediators of perceived social support and the sense of school belonging

In consistent with hypothesis 2, our study found the association between resilience and PSU was mediated by both perceived social support and the sense of school belonging. The pandemic has made it harder for the youngsters to advance academically. The families and the whole community are deeply concerned and an array of measures have been implemented to secure a safe and healthy environment around the students. According to the Broaden-and-Build theory [29], having strong resilience encourages a person to seek out practical resources to overcome obstacles. In this regard, the resilient individuals would look for resources from the families and friends. With the right perceived supports, students are more likely to stay on course and rely less on online communities for consolation, which lowers the risk that they may acquire PSU [62]. Additionally, the schools have offered supports to the students in various means. The instructors in the online programs offer consolation to the students and motivate them to keep learning. Resilient children might benefit from this and feel like they are returning to school. Students who are at peace with themselves are better able to focus on their academic work and are less likely to overuse their smartphones.

### The moderator of habitual smartphone use

Supporting hypothesis 3, habitual smartphone use acted as a moderator in the association between resilience, and PSU, perceived social support, and the sense of school belonging. With habitual smartphone use increasing, each individual influence grew stronger. A resilient person is fast to adapt to changing circumstances and skilled at using the tools at hand to overcome obstacles. Therefore, such students would call the support providers utilizing the practical and multipurpose communication tool to acquire the required supplies while at home quarantine. During the pandemic, the smartphone becomes the primary and most often the only choice in this scenario. In this regard, it makes sense that the resilient person would gain more social support and the sense of school belonging with the more frequent use of intensified habitual smartphone use (such as information seeking, texting, video chatting, etc.), and the resources acquired would lessen the likelihood to develop PSU.

### Limitations and implications

There are some limitations concerning the study. First, the findings of our study were based on a cross-sectional study. In hence, future study could base on a longitudinal survey which could better clarify the causalities among the investigated variables. Second, the current sample has an unbalanced gender distribution, which might impact the results. Third, future study should involve participants from a broader population.

Despite the limitations, our study has several implications. First, our study determined the benefits of resilience for accumulating psychological assets to attenuate the severity of PSU. Families and teachers might teach the youngsters to think less negatively and concentrate more on the good aspects of life. Additionally, students should also receive instruction on how to set realistic objectives and create a positive attitude on the future. All these measures would help the students to develop a higher level of resilience. Second, the findings add to our knowledge of the benefits of routine smartphone use by showing how the devices aid the students in times of need. Therefore, rather than just prohibiting kids from using smartphones, schools should look at ways to teach them how to use them safely so that current technology may best serve their needs.

## Conclusion

The current study focused on resilience, perceived social support, the sense of school belonging, habitual smartphone use, and PSU among Chinese college students. Our research discovered the negative influence of resilience on PSU, and specifically, highlighted the mediation effects of perceived social support and the sense of school belonging. Of significance, we also found the moderation effect of habitual smartphone use in the development of PSU. Our findings provide new insight into the role that resilience plays in PSU and underline the importance of habitual smartphone use in general.

## Data Availability

The datasets used and/or analyzed during the current study are available from the corresponding author on reasonable request.
